# *Schistosoma* gallbladder polyp masquerading as a neoplasm: Rare case report and literature review

**DOI:** 10.1002/ccr3.4420

**Published:** 2021-07-09

**Authors:** Gawahir A. Ali, Wael Goravey, Issam Al‐Bozom, Muna A. Al Maslamani, Hamad Abdel Hadi

**Affiliations:** ^1^ Department of Infectious Diseases Communicable Diseases Centre Hamad Medical Corporation Doha Qatar; ^2^ Department of laboratory medicine and pathology HMC Doha Qatar

**Keywords:** gallbladder polyps, Praziquantel, Schistosomiasis

## Abstract

Schistosomiasis affecting the gastrointestinal tract is common in tropical and subtropical areas but associated polyps presenting as gallbladder pathology are rare clinical entities necessitating high clinical suspicion.

## Graphical abstract

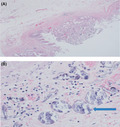

## 1 INTRODUCTION

Schistosomiasis is an ancient neglected tropical disease that mainly affecting the gastrointestinal and urogenital tracts but also capable of causing a spectrum of distant manifestations. Besides common presentations, isolated gallbladder polyps are rarely reported. We reported and reviewed the literature of a rare case of isolated gallbladder polyps in a 55‐year‐old patient.

Schistosomiasis is an ancient parasitic infection caused by the flatworm *Schistosoma* species causing primarily gastrointestinal and urogenital tract chronic pathology. There are five main subspecies but only three are clinically relevant; *Schistosoma hematobium*, *Schistosoma mansoni*, and *Schistosoma Japonicum* which when transmitted to humans lead to an acute as well as chronic disease with significant long‐term consequences.^1^ The foremost species is responsible for urogenital disease, while the latter two cause gastrointestinal tract (GIT) and hepatic disease with regional variations. The endemic disease has tropical and subtropical distribution, particularly in stagnant freshwater reserves, agricultural, and irrigation areas since the intermediate host is an aquatic freshwater snail. The disease affects around 250 million individuals with almost 800 million populations at risk leading to significant health and economic implications.^2^

Eggs released from humans’ excreta hatch and mature in species‐specific intermediate freshwater snails. Subsequently, released motile cercariae penetrate the skin of definitive human host when in contact with water than mature into adult worms that passes through distal blood vessels of lower GIT and urogenital tracts. Following disease acquisition, early acute symptoms are caused by immediate immunological reactions while chronic ones are secondary to mechanical injuries from secreted sharp eggs or immune‐mediated complications such as granulomatous inflammation and secondary fibrosis in targeted organs impeding normal functions.^1^

Since the parasite navigates organ‐specific blood vessels and secretes its pathological eggs in distal tributaries of affected organs, many organs besides the gastrointestinal or urogenital tracts are affected through local and hematogenous spread such as the renal, respiratory or the central nervous systems.^3^ Among different manifestations of Schistosomiasis at the gastrointestinal tract, gallbladder involvement is rare with no clear evidence of contribution of the disease toward acute cholecystitis or the chronic cholelithiasis.^4^ Nevertheless, we describe a rare case of a late isolated Schistosomiasis of the gallbladder, masquerading as a neoplastic polyp, discuss clinical manifestations and management as well as review the literature of similar cases.

## 2 CASE DESCRIPTION

A 55‐year‐old man with a history of hypertension and dyslipidemia presented to the surgical outpatient clinic with an incidental gallbladder polyp discovered during assessment for mild upper abdominal pains. The patient denied any current abdominal symptoms while past medical history included previous hepatitis C infection which was treated three years previously with the sustained virological response and no recurrence. Although the patient is resident in Qatar for the last 5 years, he is originally from rural Egypt where he used to live near irrigated farming. No history of Schistosomiasis was elicited.

**FIGURE 1:**
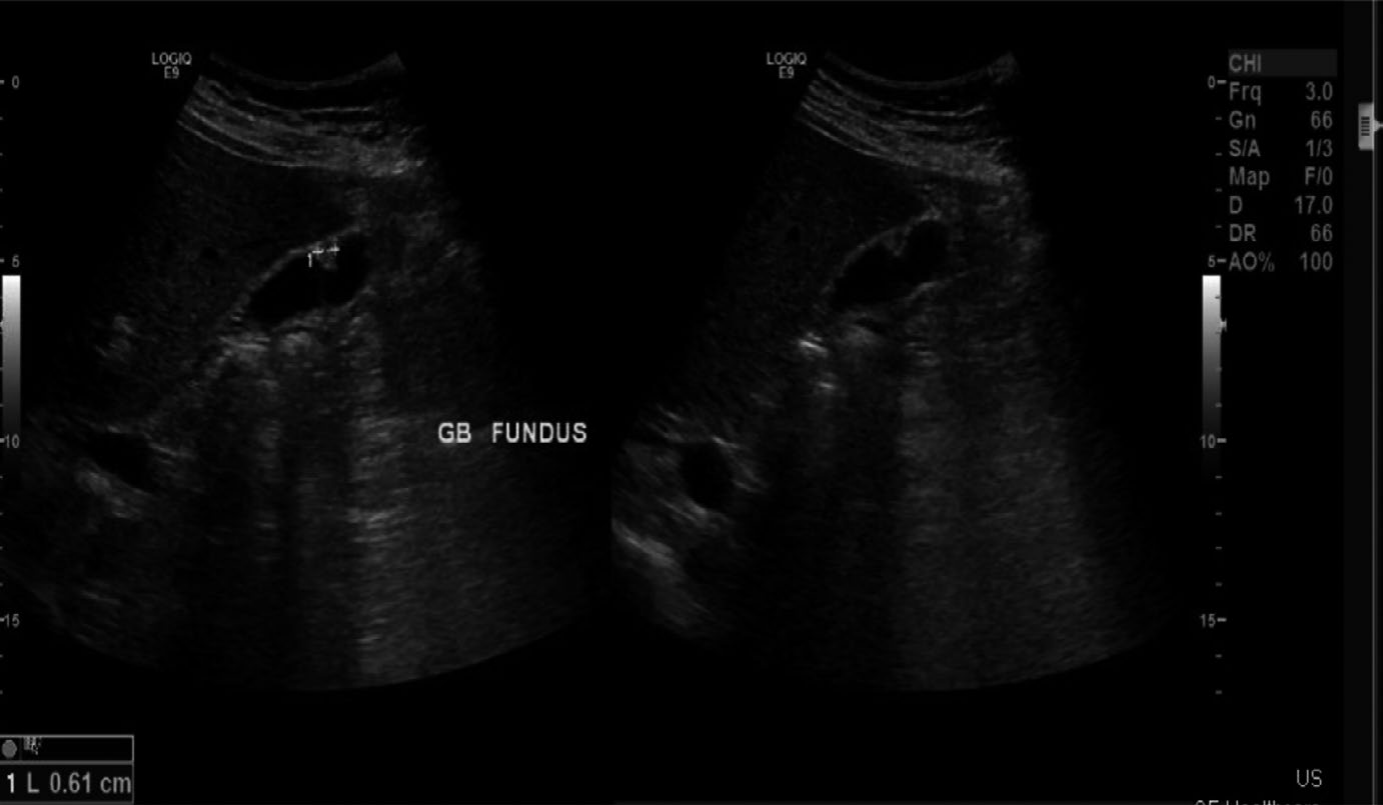
US liver and gallbladder demonstrating Gallbladder polyp in the fundus

**FIGURE 2:**
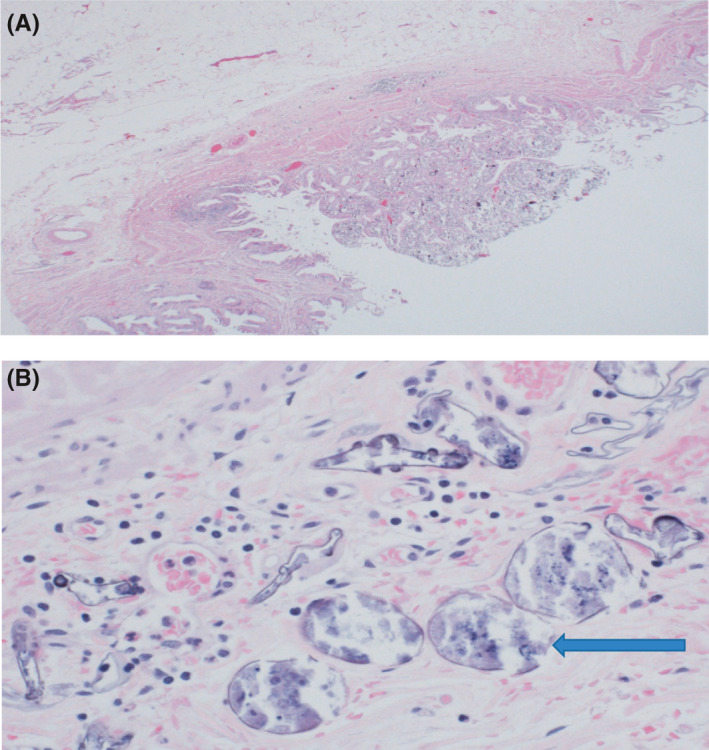
A, Light microscopic view showing polypoid projection in the lumen of the gallbladder (H&E × 20). B, Detailed examination of the polypoid projection shows numerous Schistosoma eggs (H&E × 400)

Clinical assessment was unremarkable while blood tests showed normal hemoglobin, renal, and liver functions. Repeated ultrasonography compared with previous hepatic images one year previously, demonstrated normal liver size and textures but highlighted a gallbladder polyp at the fundus measuring 6 mm growing from a previous size of 2 mm with no biliary dilatation (Figure 1). Fearing potential neoplastic pathology, the patient underwent an uneventful laparoscopic cholecystectomy to evaluate the etiology. Histopathological examination of the resected gallbladder showed a polypoid projection within the lumen of the gallbladder with abundant calcifications (Figure 2A). Close view showed numerous *Schistosoma* eggs, some of which are calcified within the mucosa, lamina propria as well as muscularis propria of the wall of the gallbladder (Figure 2B). The extensive calcification encountered in the gallbladder tissue hindered accurate speciation. Subsequent stool and urine evaluation for ova and parasites to rule out concurrent active infection were negative. Although this was considered a late complication of the primary infection, the patient was treated with Praziquantel therapy to eradicate any potential residual disease and follow up for 2 years without any new symptoms.

## 3 DISCUSSION

Schistosomiasis is an ancient parasitic disease that has evolved with humankind as evident by ancient Egyptians figurative papyrus papers.^5^ That historic link led scientists such as Theodor Bilharz to study the disease in the country during the 19th century to establish its natural course including discovering the causative organism hence equally coined Bilharzia.^6^

The disease is caused by five different *Schistosoma* species but three of clinical importance; *S mansoni*, *S japonicum*, and *S hematobium*, affecting the gastrointestinal and urogenital organs, respectively, with a complex life cycle involving species‐specific maturation phase in freshwater snails followed by infecting humans as definitive host through skin penetration when are in contact with water.^1^

The initial pathology of Schistosomiasis is caused by physical injuries of the sharp‐edged secreted eggs when passing through the lower GIT and urogenital tracts leading to the famous rectal or urinary bleeding symptoms. More importantly, immunologically mediated inflammation and local granulomata, eventually leads to secondary fibrosis replacing affected organ tissues hence normal functions.^7^ These serious long‐term complications are manifested as portal hypertension secondary to hepatic periportal fibrosis or ureteric stenosis and obstruction in the case of urogenital disease.^8^

While gastrointestinal Schistosomiasis remains one of the most clinically encountered presentations, isolated Schistosomiasis of the gallbladder is rare entity in the literature with few describe cases of secondary fibrosis and calcifications.^4^ Despite there have been postulations that the deposition of *Schistosoma* eggs at the gallbladder followed by subsequent granulomatous inflammation can lead to the common pathology of cholelithiasis or cholecystitis, no definitive association has been established.^9^

Trying to explore associative previous hepatitis C infection, the natural history of the disease is well‐established as being primarily hepatotropic leading to predominantly chronic liver disease including liver cirrhosis. Conversely, there have been observational association of increased gallbladder disease in patient with hepatitis C infection including cholelithiasis thought mainly because of secondary metabolic activity rather than the effect of therapeutic treatment.^10^ Similarly, hepatitis B rather than hepatitis C infection has been observed to be associated with gallbladder polyps in targeted population.^11^ In our case, despite the patient had previous hepatitis C infection which was adequately treated, the fact that *Schistosoma* egg was isolated from the polyp, cements being the underlying causative pathology. Of note, the extensive calcification on the tissue sample deterred the characteristic speciation based on spine of the *Schistosoma* eggs.

Our patient typically previously lived in an endemic area of Schistosomiasis in Egypt and he provided history of potential exposure of acquiring the disease through swimming in irrigation canals despite not recalling any typical symptoms. The rapidly increasing size of the gallbladder polyp during radiological assessment coupled with vague abdominal symptoms prompted the decision of operative intervention to rule out any sinister pathology, in particular, gallbladder malignancy.

Searching the literature for similar cases, yielded a total of 18 previous cases of Gallbladder schistosomiasis (Table 1). Cases ranged between 25‐77 years of age equally distributed between males and females. Cholecystitis is the predominant presentation with 11 cases associated with cholelithiasis. Most of the cases were reported from schistosomiasis endemic areas. *Schistosoma* eggs or worm was seen in the gallbladder tissues in all cases except one where details were not obtainable.^9^ Of the 18 cases reviewed, speciation from the tissue was not possible in 8 cases, though the stool gave the answer in only one of these cases.^18^ Of the 10 cases identified to *Schistosoma* species level, 6 cases were *S hematobium* and 4 cases were *S mansoni* (Table 1). Only two cases reported involvement of other organs by *Schistosoma* at the time of the diagnosis, though the data were not always available.^15^, ^17^ Similar to our case, surgery with the addition of antiparasitic treatment was implemented as management of one‐third of cases. To the best of our knowledge, only one previous case of isolated gallbladder polyps caused by Schistosomiasis is reported in the literature.^12^

**TABLE 1:** Summary of previously reported adult cases of gallbladder Schistosomiasis

Study	Case	Gender/Age, years	Nationality	Presentation	Identification of eggs/Worms in the gallbladder	*Schistosoma* species in the gallbladder tissue	Concomitant cholelithiasis	Evidences of another organ's involvement	Positive eggs in stool/urine	Management
Botha DJ,1963^13^	1	Male/27	NA	Cholecystitis	Yes	NA	Yes	Liver and bladder	NA	Surgery and anti‐bilharzial treatment.
Rappaport I,1975^14^	2	Male/51	NA	Cholecystitis	Yes	*Schistosoma mansoni*	NA	NA	NA	Surgery
M A Marcial,1989^9^	3	Female/50	NA	NA	NA	NA	NA	NA	NA	NA
al‐Saleem T,1989^15^	4‐9	4 Male, 2 Female/40.3(25‐62)	4 Iraqi/ Egyptian/ Palestine.	All Cholecystitis	Yes, all	Four *Schistosoma* *hemalobium*, one *S mansoni*, and one no speciation	5 cholelithiasis	Only in one, liver	NA	Surgery
Bakhotmah MA,1996^16^	10	male/30	NA	Cholecystitis	Yes	*S hemalobium*	Yes	No	*S hemalobium*	Surgery and praziquantel
Sharara,2001^17^	11	Female/46	Saudi	Cholecystitis	yes	*S mansoni*	No	Liver	NA	Surgery and praziquantel
Alam K,2009^18^	12‐13	Female/32, 40	NA	Cholecystitis	Yes	NA	Yes	NA	NA	Surgery
Manes K,2014^19^	14	male/77	Greek	Cholecystitis	Yes	*S mansoni*	Yes	No	No	Surgery
Daniel Azoulay,2016^20^	15	male/53	Martinique	Cholecystitis	Yes	NA	No	No	NA	Surgery and praziquantel
Majrashi SA,2018^21^	16	male/50	Saudi	Cholecystitis	Yes	*S hemalobium*	Yes	No	No	Surgery and praziquantel
Hedfi M,2019^4^	17	Female/51	Tunisian	Biliary colic	Yes	NA	Yes	No	NA	Surgery
Ghimire PG,2020^12^	18	Female/20	Nepalese	Biliary colic	Yes	NA	No, gallbladder polyp	No	*S japonicum*	Surgery and praziquantel
Our case,2021	19	male/55	Egyptian	Abdominal pain	Yes	NA	No, gallbladder polyp	No	No	Surgery and praziquantel

Abbreviation: NA, not available.

### 3.1 Conclusion

Isolated gallbladder polyps caused by Schistosomiasis are rare clinical entities, which necessitate obtaining relevant past medical history particularly in patients from endemic areas. The disease probably is a long‐term manifestation of previous exposure best diagnosed histologically.

## CONFLICT OF INTEREST

The authors declare that they have no competing interests.

## AUTHOR CONTRIBUTIONS

GA: wrote the manuscript, acquired the data, and managed clinically. WG: corresponded, contributed, and prepared the data and manuscript. IA: contributed to data acquisition and histopathology reports. HA and MA: supervised all the aspects and contributed to final manuscript editing.

## ETHICAL APPROVAL

Ethics approval and permission was obtained to publish the case reports from the institutional review board which is in line with international standards,

## Data Availability

The authors confirm that the datasets supporting the findings of this case are available from the corresponding author upon request.

## References

[1] Colley DG , Bustinduy AL , Secor WE , King CH . Human schistosomiasis. Lancet. 2014;383(9936):2253‐2264. 24698483 10.1016/S0140-6736(13)61949-2 PMC4672382

[2] Nelwan ML . Schistosomiasis: life cycle, diagnosis, and control. Curr Ther Res Clin Exp. 2019;91:5‐9. 31372189 10.1016/j.curtheres.2019.06.001 PMC6658823

[3] Schwartz C , Fallon PG . Schistosoma “Eggs‐iting” the host: Granuloma formation and egg excretion. Front Immunol. 2018;9:2492. 30459767 10.3389/fimmu.2018.02492 PMC6232930

[4] Hedfi M , Debaibi M , Ben IS , Chouchen A . Gallbladder schistosomiasis: Rare but possible, a case report and review of the literature. Pan Afr Med J. 2019;32:91. 31223382 10.11604/pamj.2019.32.91.17907 PMC6561011

[5] Di Bella S , Riccardi N , Giacobbe DR , Luzzati R . History of schistosomiasis (bilharziasis) in humans: from Egyptian medical papyri to molecular biology on mummies. Pathog Glob Health. 2018;112(5):268‐273. 30016215 10.1080/20477724.2018.1495357 PMC6225400

[6] Barnett R . Schistosomiasis. Lancet. 2018;392(10163):2431. 30527408 10.1016/S0140-6736(18)33008-3

[7] McManus DP , Bergquist R , Cai P , Ranasinghe S , Tebeje BM , You H . Schistosomiasis—from immunopathology to vaccines. Semin Immunopathol. 2020;42(3):355‐371. 32076812 10.1007/s00281-020-00789-x PMC7223304

[8] McManus DP , Dunne DW , Sacko M , Utzinger J , Vennervald BJ , Zhou XN . Schistosomiasis. Nat Rev Dis Prim. 2018;4(1):13. 30093684 10.1038/s41572-018-0013-8

[9] Marcial MA , Marcial‐Rojas RA . Cholecystitis due to schistosoma mansoni, fact or fancy. Bol Asoc Med P R. 1989;81(5):178‐179. 2500133

[10] Wijarnpreecha K , Thongprayoon C , Panjawatanan P , Lekuthai N , Ungprasert P . Hepatitis C virus infection and risk of gallstones: A meta‐analysis. J Evid Based Med. 2017;10(4):263‐270. 29193901 10.1111/jebm.12277

[11] Lin WR , Lin DY , Tai DI , et al. Prevalence of and risk factors for gallbladder polyps detected by ultrasonography among healthy Chinese: Analysis of 34 669 cases. J Gastroenterol Hepatol. 2008;23(6):965‐969. 17725602 10.1111/j.1440-1746.2007.05071.x

[12] Ghimire PG , Ghimire P . Gallbladder schistosomiasis – a rare presentation as gallbladder polyp: a case report. Radiol Case Rep. 2020;15(8):1394‐1397. 32636981 10.1016/j.radcr.2020.06.013 PMC7329932

[13] Botha DJ . Bilharzial cholecystitis with calculus. Br J Surg. 1963;50(223):543‐544. 10.1002/bjs.18005022318

[14] Rappaport I , Albukerk J , Schneider IJ . Schistosomal cholecystitis. Arch Pathol Lab Med. 1975;99(4):227‐228. 1167781

[15] Al‐Saleem T , Al‐Janabi T . Schistosomal cholecystitis: report of six cases. Ann R Coll Surg Engl. 1989;71(6):366‐367. 2513766 PMC2499044

[16] Bakhotmah MA . Gallbladder Bilharziasis. HPB Surg. 1996;9(3):175‐177. 8725460 10.1155/1996/76282 PMC2443086

[17] Sharara AI , Abi‐Saad G , Haddad M , Mansour A , Tawil A . Acute granulomatous schistosomal cholecystitis. Eur J Gastroenterol Hepatol. 2001;13(8):1001‐1003. 11507373 10.1097/00042737-200108000-00025

[18] Alam K , Maheshwari V , Jain A , et al. Schistosomiasis: a case series, with review of literature. Int J Infect Dis. 2008;7:1.

[19] Manes K , Chatzimargaritis K , Apessou D , Papastergiou V , Dervenis C . Granulomatous cholecystitis in a patient with schistosoma mansoni infection: A case report. Int J Case Rep Images. 2014;5(6):439‐443.

[20] Azoulay D , Djabbari M , Calderaro J . An unusual cause of cholecystitis. Gastroenterology. 2016;150(5):e3‐e4. 10.1053/j.gastro.2015.12.009 27025679

[21] Majrashi SA , Al Amoodi OM . Schistosomiasis as a cause of acute cholecystitis. Saudi Med J. 2018;39(7):725‐728. 29968897 10.15537/smj.2018.7.22018 PMC6146260

